# Mathematical Models for Quantitative Assessment of Bioluminescence Resonance Energy Transfer: Application to Seven Transmembrane Receptors Oligomerization

**DOI:** 10.3389/fendo.2012.00104

**Published:** 2012-08-28

**Authors:** Luka Drinovec, Valentina Kubale, Jane Nøhr Larsen, Milka Vrecl

**Affiliations:** ^1^Aerosol d. o. o.Ljubljana, Slovenia; ^2^Institute of Anatomy, Histology and Embryology, Veterinary Faculty, University of LjubljanaLjubljana, Slovenia; ^3^Department of Incretin Biology, Novo Nordisk A/SGentofte, Denmark

**Keywords:** 7TMRs, BRET, oligomerization, mathematical models, quantitative analysis

## Abstract

The idea that seven transmembrane receptors (7TMRs; also designated G-protein coupled receptors, GPCRs) might form dimers or higher order oligomeric complexes was formulated more than 20 years ago and has been intensively studied since then. In the last decade, bioluminescence resonance energy transfer (BRET) has been one of the most frequently used biophysical methods for studying 7TMRs oligomerization. This technique enables monitoring physical interactions between protein partners in living cells fused to donor and acceptor moieties. It relies on non-radiative transfer of energy between donor and acceptor, depending on their intermolecular distance (1–10 nm) and relative orientation. Results derived from BRET-based techniques are very persuasive; however, they need appropriate controls and critical interpretation. To overcome concerns about the specificity of BRET-derived results, a set of experiments has been proposed, including negative control with a non-interacting receptor or protein, BRET dilution, saturation, and competition assays. This article presents the theoretical background behind BRET assays, then outlines mathematical models for quantitative interpretation of BRET saturation and competition assay results, gives examples of their utilization and discusses the possibilities of quantitative analysis of data generated with other RET-based techniques.

## Introduction

Seven transmembrane receptors form the largest and an evolutionarily well conserved family of cell surface receptors, with more than 800 members identified in the human genome. They are the targets both for a plethora of endogenous ligands (e.g., peptides, glycoproteins, lipids, amino acids, nucleotides, neurotransmitters, odorants, ions, and photons) and therapeutic drugs, and they transduce extracellular (ECL) stimuli into intracellular (ICL) responses mainly via coupling to guanine nucleotide binding proteins (G-proteins; McGraw and Liggett, 2006). These receptors are characterized by seven α-helices, which serve as transmembrane spanning domains (TMs) that are connected by three ECL and three ICL loops. The amino (N)-terminal fragment is ECL and the carboxyl (C)-terminal tail is ICL. This common structural topology was resolved by the three-dimensional crystal structure of individual 7TMR members (reviewed by Salon et al., 2011). In addition to their well-established ligands and G-proteins, these receptors can interact with a diverse set of protein partners, including G-protein coupled receptor kinases (GRKs), adaptor proteins such as beta-arrestins, scaffolding proteins that assemble and localize receptor-signaling complexes in specific cell membrane microdomains, as well as with each other/other receptor members, thereby forming homo-/heteromeric complexes (reviewed by Maurice et al., 2011). The specificity of agonist-promoted receptor interactions with protein partners such as GRK2 (Hasbi et al., 2004; Jorgensen et al., 2008) and β-arrestins (Angers et al., 2000) is not in doubt and the 7TMR/β-arrestin interaction has been successfully exploited to develop new bioluminescence resonance energy transfer (BRET)-based screening platforms (Bertrand et al., 2002; Vrecl et al., 2004, 2009; Hamdan et al., 2005; Heding and Vrecl, 2011). In contrast, 7TMR homo-/heteromeric complexes are more difficult to investigate, since these interactions are in general constitutive and ligand-independent.

## Methodological Approaches to Studying 7TMRs Dimerization

Indirect evidence of receptor self-association already existed in the 1970s, before they were even shown to be seven transmembrane receptors (7TMRs). Following classical radioligand studies on the insulin receptor (De Meyts et al., 1973), negative cooperativity, for which dimerization is a prerequisite, was demonstrated for the β_2_-adrenergic receptor (β_2_-AR; Limbird et al., 1975) and thyrotrophin-stimulating hormone (TSH) receptor (De Meyts, 1976). In 1982, the receptor mosaic hypothesis was formulated, which proposed the functional importance of clustered receptors organized by receptor–receptor interaction (Agnati et al., 1982). Additional evidence supporting 7TMR dimerization/oligomerization was provided in the 1970s and 1980s by the use of radiation inactivation, photo-affinity labeling, cross-linking, and gel filtration methods (reviewed by Szidonya et al., 2008). Trans-complementation studies were subsequently introduced (Maggio et al., 1993a,b) in which co-expression of two non-functional mutant/chimeric receptors resulted in a gain of function. Co-immunoprecipitation, which was first utilized to study β_2_-AR dimerization (Hebert et al., 1996), was then the most frequently used method for detecting 7TMRs dimerization. Despite apparent receptor dimerization/oligomerization, there were concerns that higher order structures might be non-specific aggregations following detergent extraction of proteins from cells and membranes (reviewed by Milligan and Bouvier, 2005). Another less frequently utilized method for studying 7TMRs dimerization is sandwich ELISA (Biebermann et al., 2003; Rediger et al., 2009). The first widely accepted demonstration of 7TMR hetero-dimerization came from GABA_B_ receptors, which exclusively function in a heteromeric form (White et al., 1998). Atomic force microscopy also demonstrated an oligomeric arrangement of rhodopsin and opsin in the form of large paracrystalline arrays, which showed receptors organized into rows of dimers (Liang et al., 2003; Fotiadis et al., 2004). Again, though, it has been suggested that the observed structure is an artifact of the preparation process (Chabre et al., 2003; Chabre and le Maire, 2005). Several functional studies have also reported co-internalization and modulation of the signaling activity of hetero-dimers/-oligomers, supporting the concept of receptor oligomerization (Terrillon and Bouvier, 2004). The introduction of biophysical techniques based on resonance energy transfer (RET), such as FRET and BRET, were then needed for taking the subject of 7TMRs oligomerization to the fore of 7TMRs research, since they enable the detection of protein–protein interactions in live cells and in real-time (reviewed by Pfleger and Eidne, 2005). BRET was first used to demonstrate β_2_-adrenergic receptor (β_2_-AR) dimerization (Angers et al., 2000) and BRET-based information about 7TMRs homo-/heterodimerization has been rapidly accumulating since then (for recent reviews see Gurevich and Gurevich, 2008a,b; Kubale et al., 2008; Ferre et al., 2009; Ayoub and Pfleger, 2010; Ferre and Franco, 2010; Palczewski, 2010; Achour et al., 2011). Over 20 different biochemical and biophysical methods that have been utilized in 7TMRs oligomerization studies were recently reviewed by Kaczor and Selent (2011). This review gives a short overview of BRET technology development and then discusses the possibilities of quantitative analysis of generated data.

## BRET Principle and BRET Technology Development/Optimization

**Figure 1:**
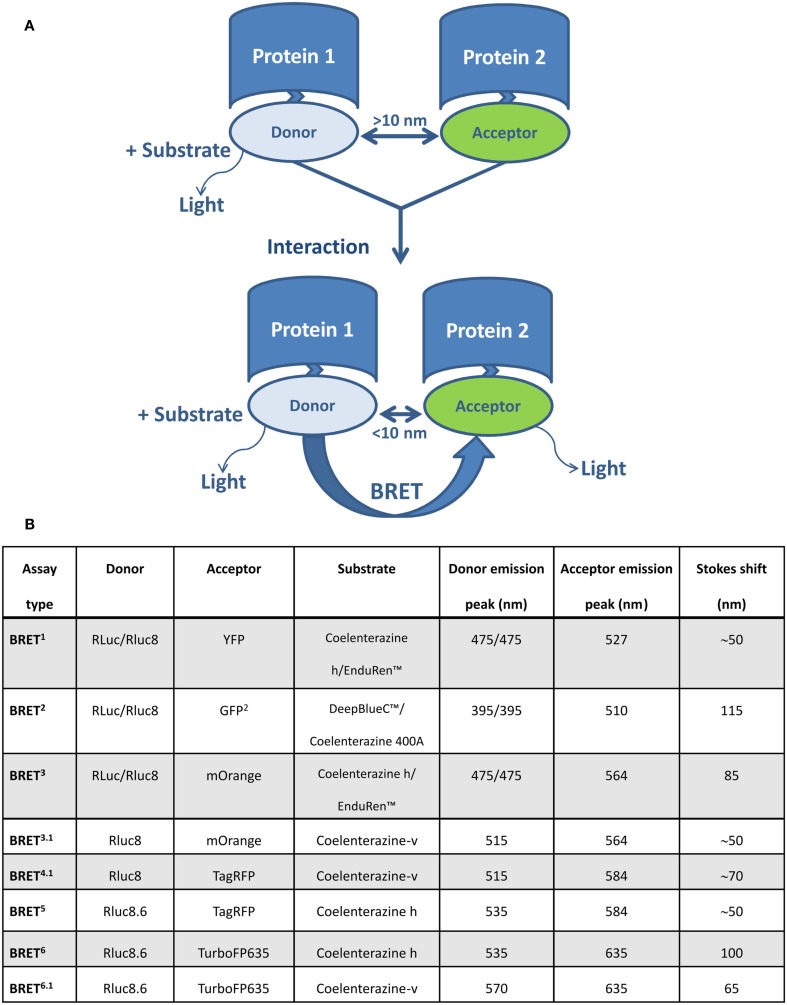
**Schematic representation of the BRET assay and various BRET variants for studying protein–protein interaction**. **(A)** BRET enables monitoring of physical interactions between two proteins genetically fused to donor and acceptor molecules. The BRET donor is a bioluminescent enzyme (a version of *Renilla luciferase*, Rluc), which reacts with the substrate to produce excitation. The acceptor molecule is usually a version of a green fluorescent protein (GFP). If the distance between donor and acceptor is more than 10 nm, light is emitted with an emission spectra characteristic for Rluc. When the distance is less than 10 nm, part of this energy is non-radiatively transferred by RET from donor (Rluc) to acceptor (version of GFP), resulting in an additional signal emitted by the acceptor. **(B)** A summary of BRET variants and their basic characteristics.

Bioluminescence resonance energy transfer enables the monitoring of physical interactions between two proteins fused to a BRET donor or acceptor moieties, depending on their intermolecular distance (1–10 nm) and relative orientation due to the dipole-dipole nature of the RET mechanism (Förster, 1959). The BRET donor is a bioluminescent enzyme (a version of *Renilla luciferase*, Rluc), which reacts with the substrate to produce excitation. Part of this excitation can be non-radiatively transferred by RET to the acceptor molecule, usually a version of the green fluorescent protein GFP (Figure 1A). In addition to the original BRET^1^ technology (Xu et al., 1999, 2003), which is based on Rluc as a donor and YFP as an acceptor, several versions of BRET assays have been developed that use different substrates and/or energy donor/acceptor pairs (Figure 1B). In BRET^2^, Rluc is used as the donor (emission peak 395 nm for coelenterazine analog DeepBlueC^™^) and GFP variant two (GFP^2^) as the acceptor molecule (excitation/emission peaks at 400/510 nm). BRET^2^ enables superior separation of donor and acceptor emission peaks (Stokes shift of 50 and 115 nm for BRET^1^ and BRET^2^, respectively), as well as efficient filtration of the excitation light, thereby enabling detection of the weak fluorescence signal. The major disadvantage of BRET^2^ compared to BRET^1^ is the 100–300 times lower intensity of emitted light and its very fast decay (Heding, 2004). This was improved by the development of suitably sensitive instruments (Heding, 2004) and the use of Rluc mutants with improved quantum efficiency and/or stability (e.g., Rluc8, Rluc8.6, and Rluc-M) as donor (De et al., 2007; Loening et al., 2007). The use of BRET^1^ and BRET^2^ is largely limited to *in vitro* cell culture systems because they emit light in the green to yellow region of the visible spectrum (510–570 nm), which is strongly absorbed by biological tissues such as blood and highly vascularized tissue. This was overcome by BRET^3^, which combines Rluc8 with the mutant red fluorescent protein (DsRed2) variant mOrange and coelenterazine or EnduRen^™^ as a substrate (De et al., 2009). In BRET^3^, the donor spectrum is the same as in BRET^1^, and the red shifted mOrange acceptor signal has excitation/emission peaks at 480/564 nm. Due to tissue attenuation of the light emitted at a wavelength <600 nm, its utilization in live animals is limited to superficial locations (e.g., subcutaneous tumors). Recently developed BRET^3^ variants (BRET^4^, BRET^5^, and BRET^6^), which have been optimized for deep-tissue imaging, combine Rluc8/Rluc8.6 with two red fluorescent proteins, i.e., TagRFP (emission peaks at 584 nm) or TurboFP635 (emission peak at 635 nm) and coelenterazine or its synthetic derivative (coelenterazine-v) as a substrate (Dragulescu-Andrasi et al., 2011).

## BRET Results – Interpretation and Possible Shortcomings

The distance (1–10 nm) at which BRET typically occurs is comparable with the dimensions of most biological macromolecules engaged in complex formation or conformational changes, thus making this technique suitable for monitoring protein–protein interactions in living cells/organisms (Wu and Brand, 1994). The experimentally determined Förster distance *R*_0_, which leads to 50% of energy transfer from the donor to the acceptor, is 4.4 and 7.5 nm for BRET^1^ and BRET^2^, respectively (Dacres et al., 2010). Even though the working distance of BRET^1^ is comparable with FRET (4.4 vs. 4.8 nm; Evers et al., 2006), the selection of RET systems depends on the particular application. FRET, under microscopic observation, allows visualization of protein interactions in living cells at the subcellular level, while BRET might be more suitable for non-imaging applications, such as the dynamic study of protein–protein interactions in a cell population (Boute et al., 2002). Direct comparison of BRET^2^ with both FRET and BRET^1^ showed the superiority of BRET^2^ over FRET and BRET^1^ in proximity-based assays such as protein–protein interaction assays (Dacres et al., 2009a,b). The working distance of 7.5 nm determined for BRET^2^ could also make it more suitable for the study of larger proteins and/or multiprotein complexes, including 7TMR complexes (Dacres et al., 2010). For comparison, the 7TMR transmembrane core spans ~4 nm across the ICL surface (Palczewski et al., 2000), the intradimer distance between rhodopsin monomers is 3.5 nm and the distance between rhodopsin dimers is 4.5 nm (Fotiadis et al., 2004). In spite of that, the following limitations of this method need to be considered when interpreting BRET results. Firstly, the size of fluorescent proteins (~27 kDa) and Rluc (~34 kDa) is comparable to that of the transmembrane core of 7TMRs (diameter ~4 nm). These proteins are usually attached to the receptor C-terminus, which varies in length in different 7TMRs from 25 to 150 amino acids. Polypeptides of this length in extended conformation can cover 8-48 nm. A BRET signal thus indicates that the donor and acceptor moieties are at distance less than 10 nm, which may occur when receptors form a structurally defined dimer or when they are >50 nm apart (reviewed by Gurevich and Gurevich, 2008a). The use of acceptor and donor molecules genetically fused to 7TMRs can alter the functionality of the receptor; fusion proteins can also be expressed in ICL compartments, making it difficult to demonstrate that the RET results from a direct interaction of proteins at the cell surface. The use of fusion proteins can therefore be a major limitation for this application. Secondly, quantitative BRET measurements are limited by the quality of the signal and noise level. Fluorescent proteins and luciferase yield background signals arising from incompletely processed proteins inside the cell and high cell autofluorescence in the spectral region used (Gurevich and Gurevich, 2008a). Thirdly, so called bystander BRET results from frequent encounters between over expressed receptors and has no physical meaning (Kenworthy and Edidin, 1998; Mercier et al., 2002). Bystander BRET is also problematic when the studied proteins are confined to a subcellular compartment, such as the plasma membrane. BRET assays should therefore be able to discriminate between true dimerization/oligomerization and random collision due to over-expression. To determine the specificity of a BRET signal, the following experiments have been proposed: negative control with a non-interacting receptor or protein, BRET saturation, and competition assays, as well as experiments that observe ligand-promoted changes in BRET (Ayoub and Pfleger, 2010; Ferre and Franco, 2010; Achour et al., 2011). Correct execution of these experiments requires graded control of protein expression over a broad range of concentration, its accurate measurement, and knowledge about the subcellular location of the expressed proteins. The choice of appropriate control is also crucially important. These requirements are not always easy to fulfill in practice and this further complicates the interpretation of results. A general method (third-party BRET), which does not require graded expression or quantification of acceptors or donors, has also been developed to detect specific constitutive BRET between proteins located in subcellular compartments of living cells; again, though, it has the same limitations as other RET methods (Kuravi et al., 2010). Additionally, proper interpretation of BRET results also requires quantitative analysis of the result, which has so far only been done in a small number of studies (Ayoub et al., 2002; Mercier et al., 2002; Vrecl et al., 2006). The theoretical background of the assays described below could serve as a guiding principle for the quantitative extrapolation of data from BRET experiments performed with 7TMRs and, presumably, also with other cell surface receptors that form dimer/oligomers.

### BRET measurement

In BRET experiments luminescence is measured at the peaks of donor and acceptor emissions (Xu et al., 1999). Given that a fixed number of quanta are produced by luciferin-luciferase reactions, the majority of light is emitted by the donor molecules. If RET takes place, then part of the energy is transferred to the acceptor molecules and is emitted at a longer wavelength. Since part of the donor emission spectra overlaps with the acceptor emission spectra, this background has to be subtracted; the BRET signal is then calculated as:

(1) $$\text{BRET = }\frac{I_{\text{2}}}{I_{\text{1}}}-\frac{I_{\text{20}}}{I_{\text{10}}},$$

where *I*_2_ and *I*_1_ are measured luminescences at the two peak positions in the case of donor-acceptor interactions, and *I*_10_ and *I*_20_ represent luminescence intensities at the two peak positions for donor only transfected cells. Samples with different concentrations of donors or variations in light quanta produced by the luciferin-luciferase reaction can in this way be compared.

In order to obtain a correct BRET value that can be compared between different laboratories, the BRET luminometer should be properly calibrated, which means that the same proportion of donor (*I*_1_) and acceptor (*I*_2_) emissions is detected. The sum of the two emissions is then proportional to the concentration of donors, which is again proportional to the total luminescence *I*_tot_ (luminescence measured in the whole visible spectrum). In general, the magnitude of measured luminescence depends on the selection of emission filters and detector sensitivity. A calibration factor *b* is thus introduced:

(2) $$I_{1}+b*I_{2}=k*I_{\text{tot}}.$$

Using the above equation, the value of parameter *b* can be assessed by completing two experiments with different donor-acceptor ratios while measuring *I*_1_, *I*_2_, and *I*_tot_. The calibrated BRET is then:

(3) $$\text{BRE}{\text{T}}_{\text{cal}}\text{ = }b*\left(\frac{I_{\text{2}}}{I_{\text{1}}}-\frac{I_{\text{20}}}{I_{\text{10}}}\right).$$

For conducting BRET assays, information about relative donor and acceptor concentrations is needed. Donor and acceptor concentrations can be assessed by luminescence and fluorescence measurements, respectively. First, however, the calibration curves of luminescence and fluorescence vs. receptor concentration must be obtained by using an immuno-based method or radioligand binding assay (Ayoub et al., 2002; Mercier et al., 2002; Ramsay et al., 2004).

### Basic BRET theory

Bioluminescence resonance energy transfer is defined as the ratio between transferred *T* and not-transferred energy *Q*:

(4) $$\text{BRET}=\frac{T}{Q}.$$

The probability that excitation is transferred from donor to acceptor in a single BRET pair is governed by the energy transfer efficiency *E*:

(5) $$T=E*Q_{0},$$

where *Q*_0_ is total energy (*Q*_0_ = *T* + *Q*). The energy transfer efficiency is inversely proportional to the sixth power of the distance *R* between the donor and the acceptor, as described by the Förster equation (Förster, 1959), where the Förster radius *R*_0_ depends on the spectral overlap and dipole orientations:

(6) $$E=\frac{R_{0}^{6}}{R_{0}^{6}+R^{\text{6}}}.$$

The calculations in a quantitative BRET assay are derived from the Veatch and Stryer model (Veatch and Stryer, 1977) covering FRET experiments with gramicidin dimers. The calculations are usually simplified by assuming that *E* is small enough for the following approximation to be used:

(7) $$\text{BRE}{\text{T}}_{E\ll 1}=\frac{T}{Q_{\text{0}}}.$$

Before using the small energy transfer approximation, the magnitude of the energy transfer efficiency *E* must be determined. For dimers, *E* can be calculated from maximum BRET, which is obtained when all donor molecules are accompanied by acceptors (using Eqs 4 and 5):

(8) $$E=\frac{\text{BRE}{\text{T}}_{\text{max}}}{\text{BRE}{\text{T}}_{\text{max}}\text{ + 1}}.$$

## Quantitative BRET Assays

Although determination of the BRET signal is quite simple, the interpretation of results obtained from oligomerization studies is not unambiguous. If the receptor expression level is in the physiological range, there is a problem of distinguishing random collisions of donors and acceptors from stable binding. With increasing receptor concentration, there is a higher probability of two receptors being in the range of the Förster radius and producing so called bystander BRET. Several quantitative assays have been developed to distinguish these two processes, including dilution, saturation, and competition assays, which allow an assessment of the receptor oligomerization state and relative affinities for homo- and hetero-dimer formation (Ayoub et al., 2002; Mercier et al., 2002). Interpretations of quantitative BRET assays have been summarized in several review articles (Issad and Jockers, 2006; Pfleger and Eidne, 2006; Ayoub and Pfleger, 2010; Achour et al., 2011; Kubale et al., 2012).

### BRET dilution assay

**Figure 2:**
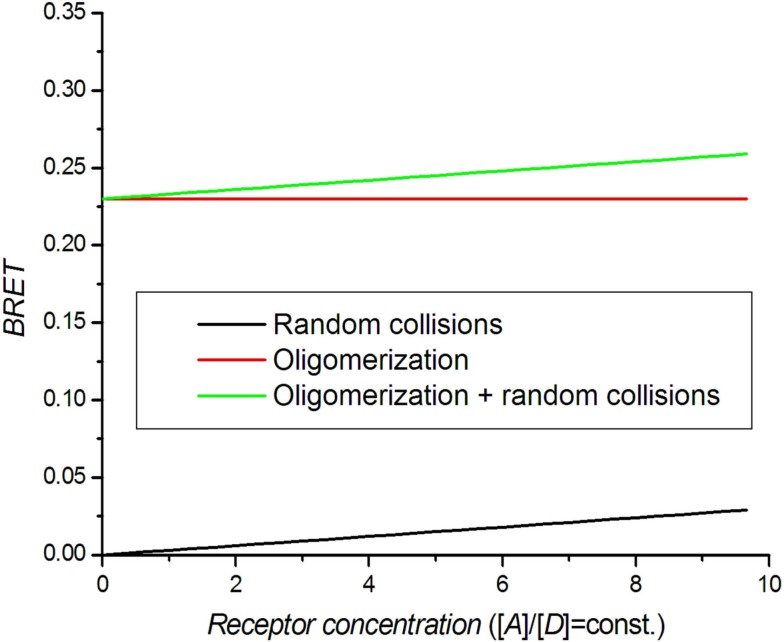
**Theoretical BRET dilution curves**. The ratio between acceptors and donors is kept constant.

A dilution assay is the simplest control experiment to check for oligomerization. RET takes place if the distance between donor and acceptor molecules is in the range of the Förster radius *R*_0_. Molecules can also get close enough for BRET by random collisions (bystander BRET) if their density is high enough (Kenworthy and Edidin, 1998; Mercier et al., 2002). Excluding random collisions, there should be no concentration dependence for coupled donor and acceptor molecules. In practice, the BRET signal can be approximated by:

(9) $$\text{BRET = BRE}{\text{T}}_{\text{0}}+k\left(\left[D\right]+\left[A\right]\right),$$

where [*D*] and [*A*] are donor and acceptor concentrations. By simultaneously lowering the concentration of both receptors (dilution), the BRET signal decreases toward BRET_0_, which is the real oligomerization signal (Figure 2). When performing this experiment, care should be taken to keep the receptor ratio [*A*]/[*D*] constant (Mercier et al., 2002).

There is a low limit of receptor concentrations that can be used in BRET assays because of the increasing noise in calculated BRET at low luminescence intensities. The upper limit of the receptor concentration used in saturation and competition assays should be set at the point at which the BRET value does not significantly differ from BRET_0_. The dilution assay has been used to set the concentration range for saturation and competition assays and to distinguish monomers from dimers (Mercier et al., 2002; Terrillon et al., 2003; Breit et al., 2004; Ramsay et al., 2004).

### BRET saturation assay

The saturation assay involves expressing a constant amount of donor-tagged receptor with an increasing amount of acceptor tagged receptor. Theoretically, the BRET signal should increase with increasing amounts of acceptor until all donor molecules are interacting with acceptor molecules. A saturation level BRET_max_ is therefore achieved, beyond which a further increase in the amount of acceptor does not increase the BRET signal (Mercier et al., 2002; Hamdan et al., 2006; Ayoub and Pfleger, 2010; Achour et al., 2011). The BRET saturation curve derived from the Veatch and Stryer model in an approximation of small energy transfer efficiency (Eq. 7) is commonly used:

(10) $$\frac{\text{BRET}}{\text{BRE}{\text{T}}_{\text{max}}}\text{ = 1}-\frac{\text{1}}{{\left(\text{1 + }\frac{\left[A\right]}{\left[D\right]}\right)}^{N}},$$

**Figure 3:**
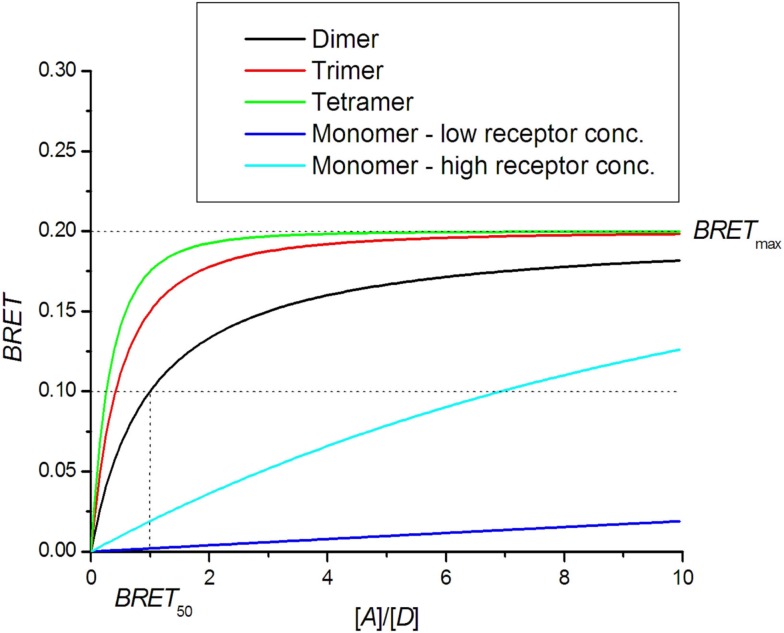
**BRET saturation assay**. Theoretical curves for oligomer formation are plotted as a function of the ratio of receptors tagged with acceptor [*A*] and donor [*D*] molecules. In the case of monomers, the BRET signal is created by random collisions.

*N *= 1 for dimer, *N *= 2 for trimer and *N *= 3 for tetramer. The detailed derivation can be found in articles by James et al. (2006), Vrecl et al. (2006). Theoretical BRET saturation curves are presented in Figure 3. BRET for higher oligomers shows faster saturation. For comparison, the monomer BRET signal that corresponds to random collisions is presented. If the receptor concentration is very high, then random collisions can generate a quasi-linear saturation curve similar to that of the dimers. A dilution experiment should thus be done first to distinguish random collisions from true oligomerization. Mercier et al. (2002) provided an equation that differs from that above for *N *> 1:

11 $$\begin{array}{c} \frac{\text{BRET}}{\text{BRE}{\text{T}}_{\text{max}}}\\ =\frac{{\left(\left[A\right]+\left[D\right]\right)}^{N+1}-{\left[A\right]}^{N+1}-{\left[D\right]}^{N+1}}{{\left(\left[A\right]+\left[D\right]\right)}^{N+1}-{\left[A\right]}^{N+1}-{\left[D\right]}^{N+1}+\left(N+1\right){\left[D\right]}^{N+1}}. \end{array}$$

For dimers, the two equations simplify to a saturation binding curve that is usually used in saturation assays:

(12) $$\frac{\text{BRET}}{{\text{BRET}}_{\max}}=\frac{\frac{\left[A\right]}{\left[D\right]}}{1+\frac{\left[A\right]}{\left[D\right]}}.$$

In Figure 3, a comparison can be made between the theoretical BRET curve for dimers and quasi-linear curve from non-specific interactions for which high BRET_max_ values can be obtained in the case of high receptor concentrations. On the other hand, the dimer curve remains insensitive to total receptor concentration. For higher oligomers, the saturation curve is shifted to the left and allows a determination of the oligomerization state (Mercier et al., 2002; Vrecl et al., 2006). The ability to determine the oligomerization state from the saturation assay is hampered by systematic errors in the determination of receptor concentrations and variations in sample treatments, resulting in a large spread of data.

BRET_50_ represents the receptor concentration ratio at which the saturation curve reaches half-maximum value. The theoretical BRET_50_ value for homo-dimers is 1. BRET_50_ values obtained for hetero-dimers can be compared with that of homo-dimers as a measure of the relative affinity for their formation. If the affinity for hetero-dimer formation is lower, the saturation curve is shifted to the right, yielding a higher BRET_50_ value (Mercier et al., 2002; Terrillon et al., 2003; Breit et al., 2004; Goin and Nathanson, 2006). In a few cases, the affinity for hetero-dimers is higher than that for homo-dimers, as shown for melatonin receptors MT_2_-MT_1_ and MT_2_-MT_2_ receptor pairs (Ayoub et al., 2004).

The BRET_max_ value can be used to detect conformational changes of the receptors forming a certain dimer (Eidne et al., 2002; Issad and Jockers, 2006). Percherancier et al. (2005) showed that ligands can cause modulation in the BRET_max_ without affecting the receptor BRET_50_ concentration ratio, revealing the change in energy transfer efficiency *E* (Eq. 5).

When using a low energy transfer approximation, it should be checked that *E* is small (*E *< 0.2) for all receptor pairs. A general formula should otherwise be used (see below).

### General BRET saturation curve

The BRET saturation curve for a general case is derived from the Veatch and Stryer model using Eq. 4 instead of Eq. 7 (Vrecl et al., 2006):

(13) $$\frac{\text{BRET}}{{\text{BRET}}_{\max}}=1-\frac{1}{E+(1-E){\left(1+\frac{\left[A\right]}{\left[D\right]}\right)}^{N}};$$

**Figure 4:**
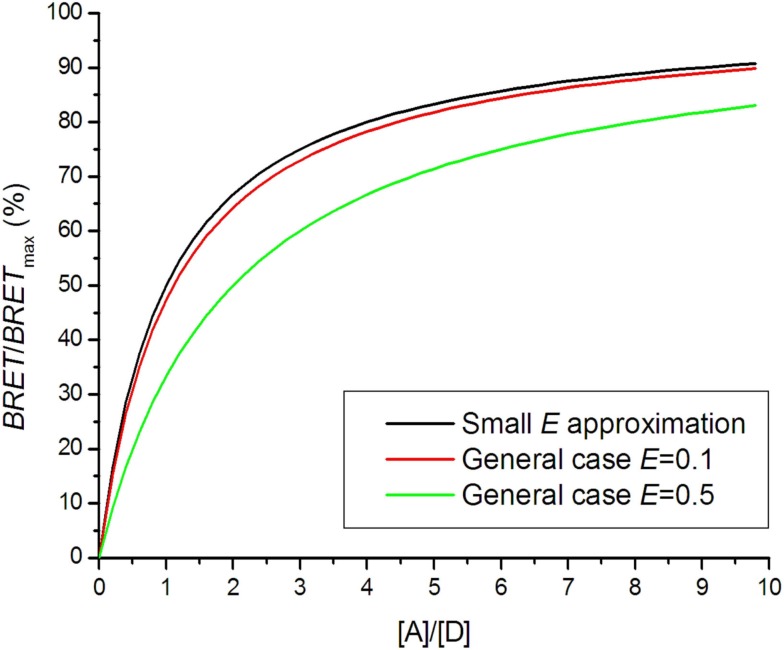
**Bioluminescence resonance energy transfer saturation curves for dimers with different energy transfer efficiencies *E***.

*N* represents the oligomerization state: *N *= 1 for dimer, *N *= 2 for trimer etc. Figure 4 shows that the saturation curve is shifted to the right for higher energy transfer efficiencies *E*, which greatly affects interpretation of the saturation assay. In several experiments using a small *E* approximation, it was observed that saturation assay data lay under the theoretical saturation curve (Mercier et al., 2002; Ramsay et al., 2004; Goin and Nathanson, 2006). The shift was interpreted as a presence of a monomeric fraction in the receptor pool, although high *E* could be responsible for the shift.

### BRET competition assay

In an attempt further to confirm the existence of oligomeric complexes, a competition assay can be performed. In this assay, the concentration of untagged receptor is increased over the concentration of donor and acceptor tagged receptors (Ayoub et al., 2002; Devost and Zingg, 2004; Vrecl et al., 2006; Achour et al., 2011). The BRET signal is expected to decrease if untagged receptors compete with tagged receptors for binding in complexes. Following the Veatch and Stryer approach (Veatch and Stryer, 1977), the BRET signal as a function of receptor concentration is obtained (Kubale et al., 2012):

(14) $$\text{BRET}=\frac{T}{Q}=\frac{E\left[\text{AD}\right]}{2\left[\text{DD}\right]+\left(1-E\right)\left[\text{AD}\right]+\left[\text{CD}\right]},$$

where [*C*] represents the concentration of untagged competitor. If all receptors form dimers and association constants are the same for AA, AD, DD, CD, AC, and CC dimers, the BRET competition curve for dimers is obtained:

(15) $${\text{BRET}}_{\text{Dimer}}=\frac{E\frac{\left[A\right]}{\left[D\right]}}{1+(1-E)\frac{\left[A\right]}{\left[D\right]}+\frac{\left[C\right]}{\left[D\right]}}.$$

The competition curve for trimers is obtained in the same way:

(16) $${\text{BRET}}_{\text{Trimer}}=\frac{2E\frac{\left[A\right]}{\left[D\right]}}{1+(1-2E)\frac{\left[A\right]}{\left[D\right]}+\frac{\left[C\right]}{\left[D\right]}}.$$

**Figure 5:**
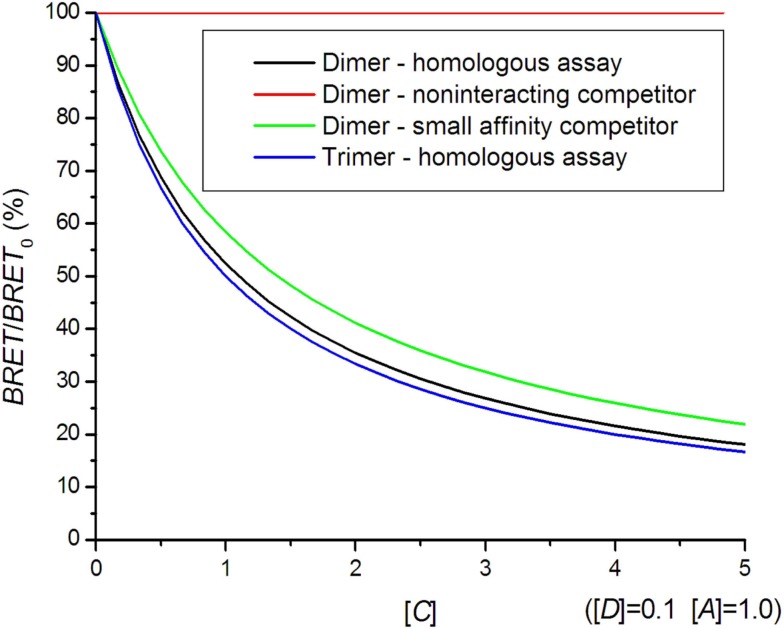
**Bioluminescence resonance energy transfer competition curves**. In a homologous assay, the same kind of receptor is used as a competitor, whereas in a heterologous assay, a different receptor with a smaller affinity for hetero-dimer formation is used.

A high acceptor to donor concentration ratio is commonly used in BRET saturation experiments, since variations in this ratio do not influence the BRET signal as much as for [*A*]/[*D*] = 1. In general, the interaction with untagged receptors causes a reduction of the BRET signal following a hyperbolic curve (Figure 5). It can very well be distinguished whether oligomerization is present but the exact oligomerization state is difficult to assess, because the dimer and higher oligomer curves are too similar. A competition assay can be used to study hetero-dimer formation. The smaller affinity for hetero-dimer formation results in a shallower competition curve (Figure 5).

In contrast to the hyperbolic competition curve (Eq. 15), a linear dependence of BRET vs. competitor concentration has been proposed for dimers (Ayoub et al., 2002). In practice, a quantitative competition assay is less frequently performed than a saturation assay due to the need to quantify the untagged competitor concentration using biochemical methods (immuno-based methods, radioligand binding). Qualitative assays using single wild type receptor concentrations have been used on several occasions to assess the specificity of the interaction (Kroeger et al., 2001; Terrillon et al., 2003; Breit et al., 2004).

### Control experiments

A non-interacting receptor should be used for negative control, which is expressed at similar levels and occupies the same cellular compartment (Terrillon et al., 2003; Pfleger and Eidne, 2005). For positive control experiments, donor and acceptor species are fused together to express a constant BRET signal. This type of experiment is used to test the measurement system and compare data from different datasets (Pfleger and Eidne, 2006).

### New RET-based techniques for oligomerization studies

In order to prove higher order oligomerization with more certainty, new RET-based approaches have been developed that combine two different techniques. A sequential-BRET-FRET (SRET) has been created that enables identification of hetero-oligomers formed by three different proteins (Carriba et al., 2008). In SRET, the oxidation of the Rluc substrate by a Rluc-fusion protein triggers excitation of the acceptor GFP^2^ by BRET^2^ and subsequent energy transfer to the acceptor YFP by FRET. SRET is the ratio between YFP and Rluc emissions. The experiment is conducted in the same way as a saturation assay, by measuring SRET with an increased YFP tagged receptor concentration.

Using the small *E* approximation and a pure trimer population, a SRET curve can be obtained in the same way as those for saturation and competition assays:

17 $$\begin{array}{c} \text{SRET}\\ =\frac{2E_{1}E_{2}\left[M\right]\left[A\right]}{{\left[D\right]}^{2}+{\left[M\right]}^{2}+{\left[A\right]}^{2}+2\left[D\right]\left[M\right]+2\left[D\right]\left[A\right]+2\left[M\right]\left[A\right]}, \end{array}$$

where [*D*] is the Rluc tagged donor, [*M*] is a GFP2 tagged “mediator” and [*A*] is the YFP tagged acceptor. If the donor and mediator concentrations are kept constant and the acceptor concentration increased, a rise toward a transient maximum and a decay toward zero for higher acceptor concentrations should be observed. In experiments performed by Carriba et al. (2008) only the rising part of the SRET curve was observed. It can be assumed that higher acceptor concentrations, for which the decaying part of the SRET curve should be observed, were not tested.

Other creative approaches to detecting receptor hetero-dimerization/multiprotein complex formation include combinations of (i) bimolecular luminescence (BiLC) and bimolecular fluorescence (BiFC), (ii) BiFC and BRET, (iii) GPCR-Heteromer Identification Technology (GPCR-HIT), and (iv) complemented donor-acceptor resonance energy transfer (CODA-RET). BiLC and BiFC enable the detection of tetramer formation (Guo et al., 2008). Complementary fragments are used to reconstitute the functional protein when brought into close proximity. A BRET signal is thus produced only in a protein complex incorporating both Rluc8 fragments, which act as donors, and both Venus fragments, which act as acceptors. By increasing the acceptor concentration, it is possible to observe the BRET concentration dependence, similar to the standard saturation curve (Eq. 12).

Bimolecular fluorescence in combination with BRET is based on the ability to produce a fluorescent complex from non-fluorescent constituents if a protein–protein interaction occurs. Two receptors are fused at their C-termini with either N-terminal or C-terminal fragments of YFP, and receptor hetero-dimerization causes YFP reconstitution. If there is heterotrimerization, BRET can then be obtained when the cells also co-express the third receptor fused to Rluc (reviewed by Ferre and Franco, 2010). GPCR-HIT utilizes BRET and ligand-dependent recruitment of 7TMR-specific interaction partners (such as a β-arrestin, PKC, or G-protein) to enable 7TMR heteromer discovery and characterization (Mustafa and Pfleger, 2011; See et al., 2011). In this set up, only one receptor subtype is fused to Rluc and the second receptor subtype is untagged. A third protein capable of interacting specifically with one or both receptors in a ligand-dependent manner is fused to the YFP. The ligand-induced BRET signal indicates that activation of the untagged receptor or the heteromer results in recruitment of the YFP tagged protein to the heteromer. The recently developed CODA-RET method combines protein complementation with RET in order to study conformational changes in response to activation of a defined 7TMR heteromer. CODA-RET quantifies the BRET between a receptor heterodimer and a subunit of the heterotrimeric G-protein. It eliminates the contribution from homodimeric signaling and enables analysis of the effect of drugs on a defined 7TMR heterodimer (Urizar et al., 2011).

## Summary

Quantitative BRET-based techniques are extremely potent tools for investigation of membrane receptor interaction in live cells and in real time, provided they are correctly conducted and data critically interpreted. A dilution assay is a basic tool for distinguishing specific binding from random interaction and is used to set the receptor concentration range for other BRET assays. Relative affinities for homo-dimer and hetero-dimer formation can be investigated using BRET competition and saturation assays. The latter can also be used to determine the oligomerization state of the receptors, if the energy transfer efficiency is known and the correct mathematical model is used. In order unambiguously to show the formation of trimers and tetramers, the use of methods that combine different RET-based techniques seems to be more suitable.

## Conflict of Interest Statement

The authors declare that the research was conducted in the absence of any commercial or financial relationships that could be construed as a potential conflict of interest.
